# End-inspiratory pause prolongation in acute respiratory distress syndrome patients: effects on gas exchange and mechanics

**DOI:** 10.1186/s13613-016-0183-z

**Published:** 2016-08-24

**Authors:** Hernan Aguirre-Bermeo, Indalecio Morán, Maurizio Bottiroli, Stefano Italiano, Francisco José Parrilla, Eugenia Plazolles, Ferran Roche-Campo, Jordi Mancebo

**Affiliations:** 1 Servei de Medicina Intensiva, Hospital de la Santa Creu i Sant Pau, Universidad Autònoma de Barcelona (UAB), Sant Quintí, 89, 08041 Barcelona, Spain; 2 Anestesia e Rianimazione 3, Ospedale Niguarda Ca’ Granda, Milan, Italy; 3 Servei de Medicina Intensiva, Hospital Verge de la Cinta, Tortosa, Spain

**Keywords:** End-inspiratory pause, Dead space, Tidal volume, Acute respiratory distress syndrome, Mechanical ventilation

## Abstract

**Background:**

End-inspiratory pause (EIP) prolongation decreases dead space-to-tidal volume ratio (Vd/Vt) and PaCO_2_. We do not know the physiological benefits of this approach to improve respiratory system mechanics in acute respiratory distress syndrome (ARDS) patients when mild hypercapnia is of no concern.

**Methods:**

The investigation was conducted in an intensive care unit of a university hospital, and 13 ARDS patients were included. The study was designed in three phases. First phase, baseline measurements were taken. Second phase, the EIP was prolonged until one of the following was achieved: (1) EIP of 0.7 s; (2) intrinsic positive end-expiratory pressure ≥1 cmH_2_O; or (3) inspiratory–expiratory ratio 1:1. Third phase, the Vt was decreased (30 mL every 30 min) until PaCO_2_ equal to baseline was reached. FiO_2_, PEEP, airflow and respiratory rate were kept constant.

**Results:**

EIP was prolonged from 0.12 ± 0.04 to 0.7 s in all patients. This decreased the Vd/Vt and PaCO_2_ (0.70 ± 0.07 to 0.64 ± 0.08, *p* < 0.001 and 54 ± 9 to 50 ± 8 mmHg, *p* = 0.001, respectively). In the third phase, the decrease in Vt (from 6.3 ± 0.8 to 5.6 ± 0.8 mL/Kg PBW, *p* < 0.001) allowed to decrease plateau pressure and driving pressure (24 ± 3 to 22 ± 3 cmH_2_O, *p* < 0.001 and 13.4 ± 3.6 to 10.9 ± 3.1 cmH_2_O, *p* < 0.001, respectively) and increased respiratory system compliance from 29 ± 9 to 32 ± 11 mL/cmH_2_O (*p* = 0.001). PaO_2_ did not significantly change.

**Conclusions:**

Prolonging EIP allowed a significant decrease in Vt without changes in PaCO_2_ in passively ventilated ARDS patients. This produced a significant decrease in plateau pressure and driving pressure and significantly increased respiratory system compliance, which suggests less overdistension and less dynamic strain.

## Background

Mechanical ventilation in patients with acute respiratory distress syndrome (ARDS) must combine both low tidal volumes (Vt) and adequate positive end-expiratory pressure (PEEP) [1, 2]. However, in patients with ARDS, respiratory acidosis and high airway plateau pressures (*P*_plat_) may limit management of ventilatory adjustments. In particular, the functional consequences of hypercapnia and respiratory acidosis may differ considerably depending on a patient’s condition, and they may involve almost any physiological function [3–6].

Optimization of mechanical ventilation parameters is associated with a reduction in dead space and is a useful strategy to reduce hypercapnia in ARDS patients [7]. Many other strategies have also been developed to decrease hypercapnia at the bedside, such as increases in respiratory rate [8], use of active humidifiers [9] and the tracheal gas insufflation [10] or aspiration of dead space [11]. At bedside, the dead space could be calculated using the Enghoff modification of the Bohr equation. The use of this equation implies the use of PaCO_2_ as surrogate for alveolar carbon dioxide. Therefore, this equation measures a global index of efficiency of gas exchange because it takes also shunt effect into account [12].

Some authors have also shown that prolonging the end-inspiratory pause (EIP) is a feasible maneuver to achieve similar targets [13, 14]. In experimental models [15] and in ARDS patients [14, 16–18], EIP prolongation has proven effective at enhancing CO_2_ elimination and decreasing partial pressure of carbon dioxide in arterial blood (PaCO_2_) and also physiological dead space (Vd_phys_). Prolonging EIP extends the time available for an enhanced diffusion between inhaled Vt and resident alveolar gas, thus facilitating the transfer of CO_2_ from alveoli toward the airways [17, 18].

Although several of the physiological studies described above have reported that EIP prolongation improves gas exchange, none have investigated the potential physiological benefits of this approach in terms of Vt reduction or improved respiratory system mechanics when hypercapnia is of no concern. To address this gap, the objective of our study was to ascertain whether EIP prolongation decreases PaCO_2_ and whether this effect can be used to decrease Vt while keeping PaCO_2_ constant. We hypothesized that this approach may have beneficial effects on respiratory system mechanics in ARDS patients.

## Methods

The study was performed in the Intensive Care Unit at Hospital de la Santa Creu i Sant Pau, Barcelona (Spain). The institutional ethics committee approved the study (Reference: 10/089), and the patients’ relatives gave signed informed consent.

### Patients

Fourteen patients who met the criteria for ARDS [19] were included in the study. Exclusion criteria were: age <18 years, pregnancy, hemodynamic or respiratory instability, and variation of more than 0.5 °C in body temperature in the last 12 h before the study was planned [20]. One patient was excluded during the study period (see Results).

All patients were under sedation and analgesia with intravenous perfusion of midazolam and opiates. Neuromuscular blockade was used in all patients to prevent triggering of the ventilator. Careful endotracheal suctioning was performed before the protocol was started. Heated humidifiers (Fisher & Paykel; MR 290 chamber and MR 850 ALU electric heater; Panmure, New Zealand) were used for airway humidification in all patients. These humidifiers were placed in the inspiratory limb of the circuit in accordance with the manufacturer’s recommendations. The respiratory rate, FiO_2_, inspiratory flow (square pattern) and PEEP were kept constant throughout the study.

### Protocol

All patients were in steady state in the 60-min preceding data recording, and all of them were in a semirecumbent position. The study was performed in three consecutive 30-min phases. Measurements in the first phase (baseline phase) were taken under the mechanical ventilation parameters set by the patient’s attending physician. In the second phase (EIP prolongation phase), the EIP was prolonged until one of the following parameters was reached: (1) EIP of 0.7 s; (2) intrinsic positive end-expiratory pressure (PEEPi) ≥1 cmH_2_O; or (3) inspiratory–expiratory ratio (I/E) of 1:1. We chose the EIP prolongation time (0.7 s) based on findings from a previous study by Devaquet et al. [18] in which a 20 % prolongation of the inspiratory time induced a significant decrease in PaCO_2_ and dead space. In the third phase (Vt reduction phase), the Vt was diminished in steps of 30 mL every 30 min until PaCO_2_ reached baseline levels.

The following data were collected at inclusion: demographic variables (age, sex, height), simplified acute physiology score II, ARDS etiology and days of mechanical ventilation.

During the last minute of each phase, we collected the following respiratory variables: peak airway pressure, Pplat, mean airway pressure, PEEPi, PEEP, driving airway pressure (∆Paw), Vt, dead space-to-Vt ratio (Vd/Vt), static compliance of the respiratory system (Crs) and airway resistance. At the same time, we recorded the following gas exchange variables: pH, partial pressure of arterial oxygen (PaO_2_), PaCO_2_ and end-tidal carbon dioxide concentration in the mixed expired gas (EtCO_2_). PEEPi was measured with a prolonged end-expiratory pause of 4 s, performed using the ventilator expiratory hold button. EtCO_2_ was measured continuously with a CO_2_ mainstream sensor (General Electric Capnostat, Milwaukee, WI, USA). The mean value of the last 10 recorded EtCO_2_ values in each phase of the study was used for analysis.

Ventilatory settings and airway pressures were recorded directly from the ventilator monitoring system. Plateau pressure was measured during an end-inspiratory pause. Dead space was calculated using the Enghoff modification of the Bohr equation [21]: Vd/Vt = (PaCO_2_ − PeCO_2_)/PaCO_2_, being PeCO_2_ the partial pressure of carbon dioxide in mixed expired gas. Expired gas was measured by collecting gas for 3 min with a Douglas bag (P-341–60; Warren E. Collins Inc., Boston, MA, USA) attached directly to the expiratory port of the ventilator. An automated analyzer (ABL 520; Radiometer A/S, Copenhagen, Denmark) was used to measure expired and arterial gases. Dead space data were expressed as physiological dead space (Vd_phys_ in mL), defined as the sum of instrumental, anatomic and alveolar dead space [22]. Driving pressure (cmH_2_O) was calculated as Pplat-PEEP. Crs (mL/cmH_2_O) was calculated as Vt/[Pplat-(PEEP + PEEPi)], and airway resistance (cmH_2_O/L/s) was calculated as (peak airway pressure − plateau pressure)/Flow. Predicted body weight (PBW) was calculated as follows: 50 + 0.91(height in cm-152.4) for men and 45.5 + 0.91(height in cm-152.4) for women [8]. Arterial to end-tidal CO_2_ gradient (P(a-et)CO_2_) was calculated in each study phase. We used Puritan Bennett™ 840 (Covidien, Galway, Ireland) and Dräger Evita XL (Dräger Medical, Lübeck, Germany) ventilators. All the ventilators used have a compressible volume compensation system.

### Statistical analysis

Data are expressed as mean ± standard deviation. The results were analyzed using one-way analysis of variance for repeated measures (ANOVA) with the Greenhouse–Geisser correction. We performed the Kolmogorov–Smirnov test to confirm normal data distribution. Since the distribution of the data was normal, we used the Student’s *t* test and the Pearson linear correlation to compare data and correlations between phases and variables, respectively. A two-tailed *p* value less than 0.05 was considered statistically significant. The SPSS^®^ Statistics (version 20.0, Chicago, IL, USA) statistical software was used for statistical analysis.

## Results

One of the 14 patients enrolled in the study was excluded from the analysis due to fever, tachypnea and unstable EtCO_2_ during the second phase of the study. The study was performed 5 ± 4 days after starting mechanical ventilation. Table 1 shows demographic data at admission, ARDS etiology and baseline characteristics at study day.

**Table 1:** Demographic data at admission and baseline characteristics of patients on the study day

Admission							Study day					
Patient	Age (years)	Gender	SAPS II	PBW (kg)	Measured weight (kg)	ARDS etiology	Days of MV before study	PaO2/FiO2 (mmHg)	FiO2a	PEEP (cmH_2_O)^a^	Flow (L/min)^a^	RR (bpm)^a^
1	75	M	59	67.7	58.5	Pneumonia	8	112	0.7	10	70	22
2	52	M	42	68.7	78	Aspiration	13	185	0.65	12	57	20
3	46	F	30	52.4	61	Multiple Trauma	7	118	0.7	12	60	25
4	62	F	69	47.9	55	Pneumonia	5	131	0.6	10	60	25
5	56	F	23	52.4	61.5	Pneumonia	3	100	0.8	12	60	22
6	66	M	40	63.2	72.5	Pneumonia	1	184	0.5	10	60	20
7	57	M	62	69.6	83	Pneumonia	1	147	0.5	8	60	17
8	36	M	24	61.4	90	Pneumonia	4	242	0.5	14	75	23
9	55	M	49	66.8	72	Pneumonia	2	219	0.6	14	70	21
10	51	F	60	43.3	64	Sepsis	12	269	0.4	8	50	21
11	74	F	61	47.9	62.5	Sepsis	1	266	0.5	10	60	21
12	43	M	61	59.6	80.5	Sepsis	3	194	0.7	10	60	22
13	63	M	30	83.1	106	Pneumonia	6	283	0.35	8	60	30
Mean ± SD	57 ± 11		47 ± 16	60.3 ± 11.2	72.6 ± 14.6		5 ± 4	188 ± 64	0.58 ± 0.13	11 ± 2	61 ± 7	22 ± 3

*ARDS* acute respiratory distress syndrome, *FiO*_*2*_ fraction of inspired oxygen, *MV* mechanical ventilation, *PaO*_*2*_ */FiO*_*2*_ partial pressure of arterial oxygen over fraction of inspired oxygen, *PBW* predicted body weight, *PEEP* positive end-expiratory pressure, *RR* respiratory rate, *SAPS II* simplified acute physiology score II

^a^These settings were kept constant throughout the study

**Fig. 1:**
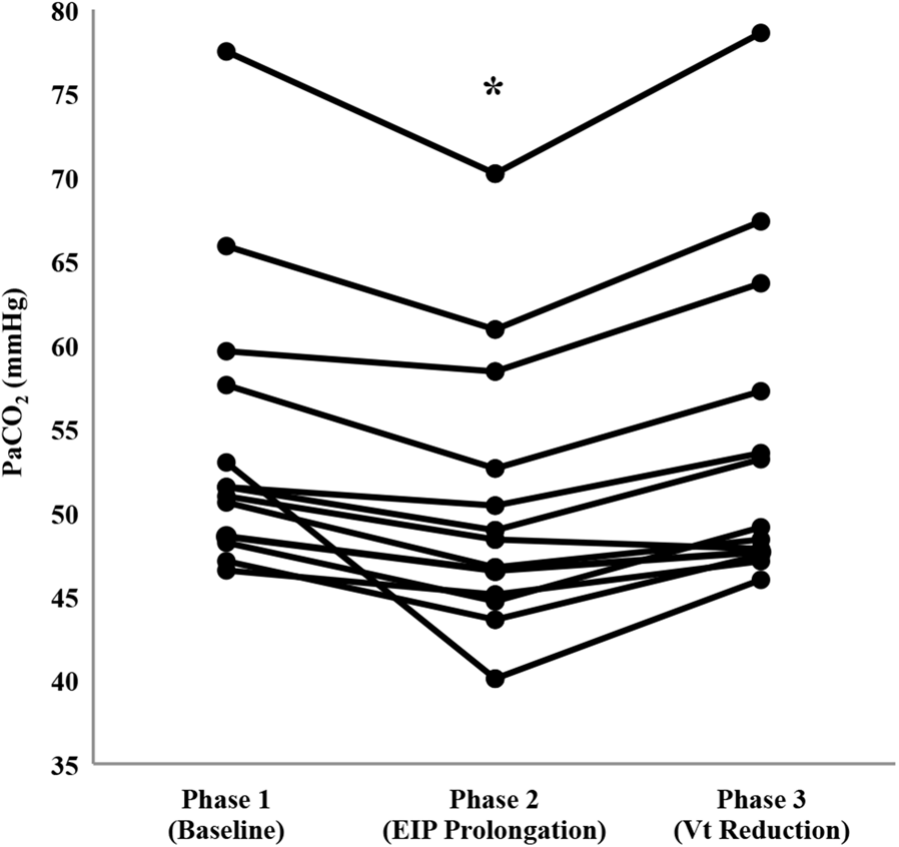
Individual values for PaCO_2_ during the study. The *asterisk* denotes statistically significant differences (*p* < 0.001) during prolongation of end-inspiratory pause. *EIP* end-inspiratory pause, *PaCO*_*2*_ partial pressure of carbon dioxide in arterial blood, *Vt* tidal volume

**Fig. 2:**
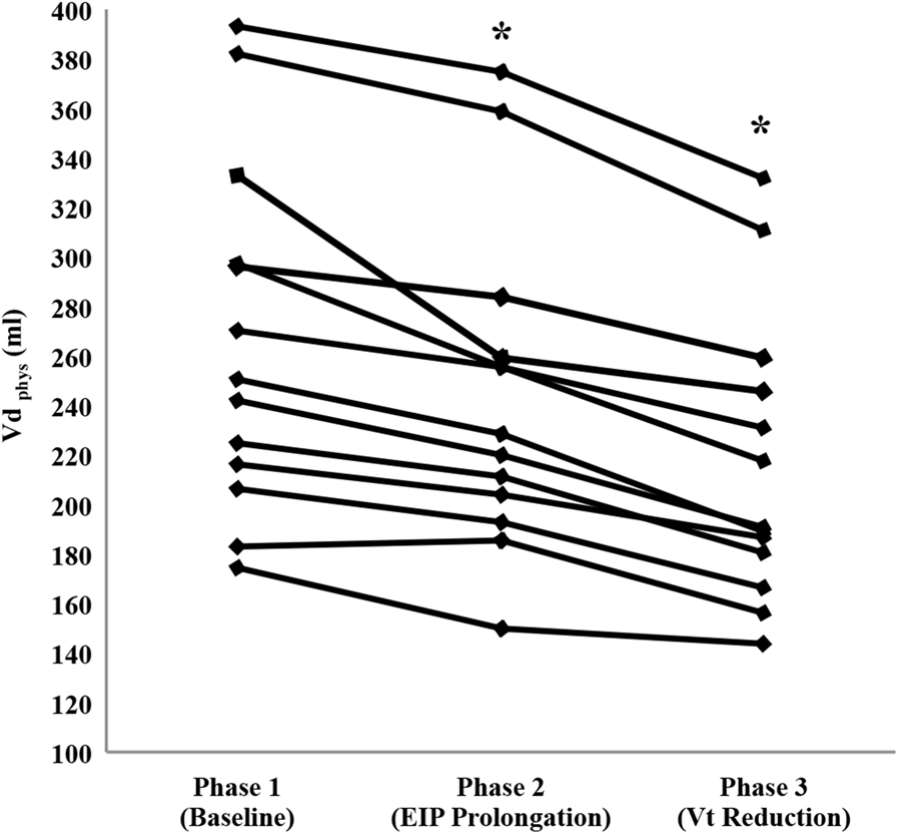
Individual values for Vd_phys_ during the study. The *asterisks* denote a significant, progressive decrease in Vd _phys_ (*p* < 0.001) during prolongation of end-inspiratory pause (EIP) and during Vt reduction. *EIP* end-inspiratory pause, *Vd*_*phys*_ physiological dead space, *Vt* tidal volume

Baseline EIP was 0.12 ± 0.04 s, and it was increased to 0.7 ± 0 s in all patients (*p* < 0.001). This EIP change was performed maintaining PEEPi <1 cmH_2_O (0.2 ± 0.2 to 0.5 ± 0.4 cmH_2_O, *p* = 0.06) and without the I/E inverse ratio ventilation (1:4.7 ± 0:1.3 to 1:1.7 ± 0:0.4, *p* = <0.001). EIP prolongation decreased Vd_phys_ and PaCO_2_ significantly with respect to basal conditions (267 ± 71 to 244 ± 65 mL and 54 ± 9 to 50 ± 8 mmHg, respectively; *p* < 0.001 for both comparisons). The decrease in PaCO_2_ levels due to EIP prolongation was correlated with the drop in Vd_phys_ (*r* = 0.871; *p* < 0.001). Individual changes in PaCO_2_ and in Vd_phys_ are shown in Figs. 1 and 2, respectively.

Between the first and second phase, significant decreases were observed in both the Vd/Vt ratio (0.70 ± 0.07 to 0.64 ± 0.08; *p* < 0.001) and EtCO_2_ (41 ± 6 to 39 ± 6 mmHg; *p* = 0.006). Basal Vd_phys_ and P(a-et)CO_2_ had a close correlation (*r* = 0.75; *p* = 0.003). The change in Vd_phys_ and the change in P(a-et)CO_2_ between the first and second phase also showed a close correlation (*r* = 0.68; *p* = 0.001).

In the third phase (EIP prolongation and Vt reduction), the Vt was significantly reduced as compared to previous phases (6.3 ± 0.8 to 5.6 ± 0.8 mL/Kg PBW; *p* < 0.001). In the third phase, as per protocol design, the PaCO_2_ and pH values were statistically identical to those at baseline (54 ± 9 vs. 54 ± 10 mmHg; *p* = 0.90 and 7.31 ± 0.07 vs. 7.31 ± 0.08; *p* = 0.90, respectively).

The Vd_phys_ decreased progressively and significantly during all phases of the study (267 ± 71 to 244 ± 65 to 216 ± 58 mL; *p* < 0.001). The Vd_phys_ and Vt at baseline were strongly correlated (*r* = 0.946; *p* < 0.001). Additionally, the Vt reduction was tightly correlated with the decrease in Vd_phys_ (*r* = 0.894; *p* < 0.001). Respiratory system mechanics, gas exchange, hemodynamics, and temperature data throughout the study are also given in Table 2.

**Table 2:** Respiratory system mechanics, gas exchange and hemodynamic data during the study

	Phase 1 (baseline)	Phase 2 (EIP prolongation)	Phase 3 (Vt reduction)	Overall *p* value	Intergroup differences
EIP (s)	0.12 ± 0.04	0.7 ± 0	0.7 ± 0	<0.001	a, b
Ppeak (cmH_2_O)	38 ± 6	38 ± 6	35 ± 5	<0.001	b, c
Pmean (cmH_2_O)	15 ± 3	18 ± 2	17 ± 2	<0.001	a, b, c
Pplat (cmH_2_O)	24 ± 3	24 ± 3	22 ± 3	<0.001	b, c
PEEPi (cmH_2_O)	0.2 ± 0.2	0.5 ± 0.4	0.5 ± 0.4	0.06	
Vt (mL)	378 ± 73	378 ± 73	336 ± 61	<0.001	b, c
Vt (PBW; mL/Kg)	6.3 ± 0.8	6.3 ± 0.8	5.6 ± 0.8	<0.001	b, c
Vd_phys_ (mL)	267 ± 71	244 ± 65	216 ± 58	<0.001	a, b, c
Vd/Vt	0.70 ± 0.07	0.64 ± 0.08	0.64 ± 0.08	<0.001	a, b
Crs (mL/cmH_2_O)	29 ± 9	29 ± 9	32 ± 11	0.001	b, c
∆ Paw (cmH_2_O)	13.6 ± 3.6	13.4 ± 3.6	10.9 ± 3.1	<0.001	a, b, c
R_aw_ (cmH_2_O/L/s)	14 ± 5	13 ± 5	13 ± 4	0.28	
pH	7.31 ± 0.07	7.34 ± 0.09	7.31 ± 0.08	<0.001	a, c
PaO_2_ (mmHg)	102 ± 23	98 ± 23	105 ± 29	0.35	
PaCO_2_ (mmHg)	54 ± 9	50 ± 8	54 ± 10	<0.001	a, c
EtCO_2_ (mmHg)	41 ± 6	39 ± 6	43 ± 7	0.002	a, c
P(a-et)CO_2_ (mmHg)	13 ± 6	12 ± 8	12 ± 9	0.27	
MAP (mmHg)	80 ± 12	76 ± 9	77 ± 12	0.08	
HR (beats/min)	87 ± 19	83 ± 20	86 ± 21	0.14	
Temperature (°C)	36.7 ± 0.9	36.7 ± 0.9	36.6 ± 0.8	0.61	

Data are presented as number (%) or mean ± SD

Intergroup differences (*p* < 0.05): a, phase 1 versus phase 2; b, phase 1 versus phase 3; c, phase 2 versus phase 3

*Crs* static compliance of the respiratory system, *EIP* end-inspiratory pause, *EtCO*_*2*_ end-tidal carbon dioxide concentration in the expired air, *FiO*_*2*_ fraction of inspired oxygen, *HR* heart rate, *MAP* mean arterial pressure, *PaO*_*2*_ partial pressure of oxygen in arterial blood, *PaCO*_*2*_ partial pressure of carbon dioxide in arterial blood, *PBW* predicted body weight, *PEEPi* intrinsic positive end-expiratory pressure, *Pmean* mean airway pressure, *Ppeak* peak airway pressure, *Pplat* plateau airway pressure, P(a-et)CO_2_ arterial to end-tidal CO_2_ gradient, *Raw* airway resistance, *Vd*_*phys*_ physiological dead space, *Vd/Vt* dead space-to-Vt ratio, *Vt* tidal volume, *∆Paw* driving airway pressure

## Discussion

The main finding of our study was that the end-inspiratory pause prolongation allowed to decrease tidal volume while maintaining similar PaCO_2_ levels. Indeed, the decrease in tidal volume led to a significant decrease in Pplat and ∆Paw, and it also improved the respiratory system compliance.

Several studies have shown that prolongation of EIP enhances CO_2_ elimination and decreases dead space and PaCO_2_ levels [14–18]. Diffusion of CO_2_ is time dependent, and EIP prolongation increases the time available for alveolar gas exchange [14, 23, 24]. Devaquet et al. [18] extended inspiratory time from 0.7 ± 0.2 to 1.4 ± 0.3 s by increasing the inspiratory pause time from 0 to 20 % of the total breathing cycle. They observed that this modification significantly decreased both Vd/Vt (around 10 %) and PaCO_2_ (around 11 %). Despite these beneficial effects of prolonged EIP and the direct relationship between inspiratory time and enhanced CO_2_ elimination [16, 18], EIP prolongation may lead to potentially adverse effects such as PEEPi production and inversion of the I/E ratio together with increases in mean airway pressure. These effects might also provoke hyperinflation, thus altering cardiac performance [25, 26]. Nevertheless, Devaquet and colleagues [18] showed that EIP could be prolonged without significantly increasing PEEPi (I/E ratio 1:1.5). Not surprisingly, and in spite of a significant increase in EIP, we did not induce any significant increase in PEEPi since the expiratory time was long enough to avoid air trapping at the end of a passive expiration (average expiratory time 1.7 ± 0.3 s). Actually (see Table 2), the average product of three time constants (the time needed to passively exhale 96 % of inhaled tidal volume) was in our patients about 1.1 s. (0.373 × 3 = 1.1 s), well below to the average expiratory time.

Prolongation of EIP in our patients caused a significant decrease in dead space and PaCO_2_ levels that was similar to previously reported [14–18]. When comparing phase 1 (baseline) and phase 2 (isolated EIP prolongation), we found that the decrease in the Vd/Vt correlated well with the drop in PaCO_2_ (*r* = 0.810; *p* < 0.001). These changes observed in our patients may be explained by the increase on the time available for distribution and diffusion of inspired tidal gas within resident alveolar gas during EIP prolongation [14]. Indeed, total PEEP levels, airflow, respiratory rate, tidal volume and respiratory mechanics were totally unchanged in this phase of our study [14, 27, 28].

Comparing the second (isolated EIP prolongation) and third (EIP prolongation and Vt reduction) phases, our data showed that the Vd/Vt ratio remained unchanged. However, the Vd_phys_, expressed in mL, decreased significantly between phases 2 and 3. This is explained by the significant reduction in Vt (that also provoked a decrease in Vd_phys_) during the third phase as compared to the previous phases, and thus Vd/Vt ratio did not change. The fact that the reduction in Vt in the third phase was accompanied by a significant decrease in Vd_phys_ and ΔPaw (with a significant increase in compliance) suggests that some degree of overdistension might be present at baseline.

As previously described, low tidal volume ventilation in ARDS may induce hypercapnia and, secondarily, induce pulmonary artery hypertension that may impair right ventricular function [29] and eventually cause acute cor pulmonale [30]. To reduce hypercapnia in ARDS ventilated patients, active heated humidifiers are often used. These devices significantly decrease dead space, PaCO_2_ and ventilator mechanical load [9] without increasing airflow resistance [31]. Although active humidification is recommended over heat and moisture exchangers in ARDS patients [32], two studies focussing on the effects of EIP prolongation on gas exchange [16, 17] did not describe the type of humidification used in their patients. A third study used passive or active humidification (10 and 5 patients, respectively) [18]. However, the effects on PaCO_2_ in all these studies [16–18] were consistently the same, thus suggesting that humidification type per se does not influence the effects of EIP on PaCO_2_.

Another technique used to decrease hypercapnia is to increase the respiratory rate. However, in ARDS patients, several studies have shown that a high respiratory rate led to gas trapping and induced PEEPi [33, 34]. In addition, experimental models suggested that higher respiratory rates may contribute to the development of ventilator-induced lung injury [35, 36]. Vieillard-Baron et al. [25] compared two respiratory rate strategies, 30 versus 15 breaths/min. They found that the high respiratory rate did not reduce PaCO_2_ levels but produced dynamic hyperinflation and reduced the cardiac index. In our patients, EIP prolongation was achieved with a relatively high inspiratory flow rate (1 L/s), thus avoiding inverse I/E ratio. This was a safe strategy to decrease PaCO_2_ levels, while keeping respiratory rate constant (22 breaths/min) and not generating PEEPi.

In our study, the reduction in Vt to maintain isocapnia was modest. Should major reductions in Vt were required, then the use of invasive extracorporeal carbon dioxide removal devices had to be considered in order to avoid acute hypercapnia [37].

Studies analyzing the EIP prolongation did not describe changes in PaO_2_ [14, 18], except one study by Mercat et al. [16]. This latter study found a slight, but not statistically significant, increase in PaO_2_ levels during EIP prolongation. This finding was not confirmed in our study. We speculate that the length of time that patients are maintained with EIP prolongation and the mean airway pressure achieved during extended EIP may have contributed to this finding. Indeed, in Mercat’s study [16], EIP prolongation was continued for 1 h with a mean airway pressure of 21 cmH_2_O and an I/E ratio 1.1. In contrast, in Devaquet’s study [18] and in our own study, EIP prolongation was shorter (30 min in both), mean airway pressure was lower (15 and 17 cmH_2_O, respectively), and the I/E ratios achieved were 1:1.5 in Devaquet’s study and 1:1.7 in ours.

The main novelty of our study is that prolonging EIP allowed to reduce Vt by 11 % (from 6.3 ± 0.8 to 5.6 ± 0.8 mL/kg of PBW; *p* < 0.001), maintaining PaCO_2_ levels equal to baseline. These sequential ventilatory changes were accompanied by a reduction in Vd_phys_. Also, when PaCO_2_ returned to baseline due to a reduction in Vt, we found a significant decrease in Pplat and an increase in Crs. In addition, these changes in ventilatory mechanics were accompanied by a significant decrease in ∆Paw. All those findings could be explained by a degree of baseline overinflation even though our initial Vt was low [38]. We further support our contention by the tight correlation between Vt and Vd_phys_ at the onset of the study and the tight correlation between the decrease in Vt and Vd_phys_ at the end of the study. Our patients were basally ventilated with parameters similar to those used in previous studies [16–18] in terms of Vt and PEEP, and Vd/Vt was also similar. Moreover, in our patients, Crs was lower (29 mL/cmH_2_O) than in Mercat and Devaquet studies (37 and 50 mL/cmH_2_O, respectively). Our findings thus suggest that if PaCO_2_ is clinically tolerable, EIP prolongation in ARDS provides physiological benefits including a small and consistent decrease in Vt which may help decrease dynamic strain [39].

In our study, a slight but not statistically significant decrease in mean arterial pressure was observed. Such trend could have been the result of complex interactions of PaCO_2_ and mean airway pressure in cardiovascular system.

We think that EIP prolongation is a feasible maneuver to optimize the consequences of mechanical ventilation in ARDS patients. Physicians may consider using an EIP prolongation in the early phase of ARDS when patients often require sedation and neuromuscular blocking agents. In our study, we have effectively implemented this strategy by using active humidification, relatively high inspiratory flow rates and close monitoring of PEEPi. This bundle decreases PaCO_2_, which in turn will allow to further decrease Vt and the consequent lung strain when isocapnic conditions are met.

One of the limitations of our study is the relatively small number of patients, the majority with pneumonia, and the fact that the study is short term. Studies with patients with different ARDS etiologies and larger numbers are warranted to confirm our data. Also, we did not measure other parameters such as inflammatory mediators or lung volumes. The calculation of dead space using the Enghoff modification of Bohr equation in patients with large shunt fractions (>20–30 %) could underestimate dead space fraction [12]. In our study, we did not measure intrapulmonary shunt. However, according to the gas exchange values that we obtained, shunt fractions above 30 % are unlikely. Additionally, the EIP prolongation increases the mechanical inflation time and it could extend into neural expiration. Asynchronies may thus develop and cause an inadequate patient–ventilator interaction when the patients are not paralyzed [39–41]. Our results could be dependent on our routine management of mechanical ventilation in ARDS patients, but our findings have been consistent in all patients and we consider they could be extrapolated to other ARDS patients. Finally, the absolute decrease in tidal volume, although statistically significant, is moderate.

## Conclusions

In conclusion, our data indicate that EIP prolongation is a simple and feasible strategy to decrease dead space and PaCO_2_ levels. In addition, when PaCO_2_ levels are of no clinical concern, EIP prolongation allows us to further decrease tidal volume. This, in turn, decreases plateau airway pressure, driving airway pressure and improves respiratory system compliance, suggesting less overdistension and less risk of dynamic strain and lung injury. Therefore, the use of this simple ventilator maneuver during mechanical ventilation in sedated and paralyzed ARDS patients merits consideration.

## Abbreviations

ARDS: acute respiratory distress syndrome
Crs: static compliance of the respiratory system
EIP: end-inspiratory pause
EtCO_2_: end-tidal carbon dioxide concentration in the mixed expired gas
IE: inspiratory–expiratory ratio
PaCO_2_: partial pressure of carbon dioxide in arterial blood
PaO_2_: partial pressure of arterial oxygen
PBW: predicted body weight
PeCO_2_: partial pressure of carbon dioxide in mixed expired gas
PEEP: positive end-expiratory pressure
PEEPi: intrinsic positive end-expiratory pressure
*P*_plat_: plateau airway pressure
P(a-et)CO_2_: arterial to end-tidal CO_2_ gradient
Vd/Vt: dead space-to-Vt ratio
Vd_phys_: physiological dead space
Vt: tidal volume
∆Paw: driving airway pressure
